# Case report: sub-clinical extramedullary B-ALL in the setting of relapse following targeted therapy

**DOI:** 10.3389/fimmu.2024.1423487

**Published:** 2024-09-25

**Authors:** Claire Johns, Courtney Erickson, Ashley Jacobs, Jennifer Moon, Christina Baggott, Regina Dagher, Helen Nadel, Jay Balagtas, Catherine Aftandilian, Sneha Ramakrishna, Norman Lacayo, Kara L. Davis, Elliot Stieglitz, Liora Schultz

**Affiliations:** ^1^ Division of Pediatric Hematology/Oncology/Stem Cell Transplantation and Regenerative Medicine, Department of Pediatrics, Stanford Medicine, Stanford, San Francisco, CA, United States; ^2^ Division of Pediatric Oncology, Department of Pediatrics, University of California San Francisco School of Medicine, San Francisco State, San Francisco, CA, United States

**Keywords:** Immunotherapy, Pediatrics, CAR (chimeric antigen receptor) T cells, B-ALL, extramedullary acute lymphoblastic leukemia, relapsed leukemia, refractory leukemia

## Abstract

Standard testing for disease evaluation in B-cell acute lymphoblastic leukemia (B-ALL) includes examination of the bone marrow and cerebrospinal fluid. Radiographic or functional imaging are indicated when clinical signs of non-CNS extramedullary disease are present but are not standard in the relapsed/refractory setting. We describe two cases of patients with relapsed/refractory B-ALL with prior exposure to blinatumomab and/or inotuzumab ozogamicin presenting for CAR-T cell treatment. Both patients were thought to only have minimal residual disease (MRD) at the pre-CAR disease assessment, with MRD of 6,648 (0.66%) and 100 (0.01%) cells per million cells, respectively, as measured by next-generation sequencing (NGS) in their bone marrows. Both patients for distinct reasons unrelated to non-CNS extra-medullary (EM) symptoms had PET-MRIs prior to lymphodepletion and CAR T cell infusion. In both cases patients were found to have significant bulky subclinical EM disease that required changes in clinical management. In the newly-emergent era of antigen-targeted immunotherapy, it is foundational that incidence and relapse patterns following targeted therapy are well-understood. Herein we contribute to a growing body of literature addressing this fundamental clinical gap and highlight a future role for formal prospective imaging studies to better establish response, toxicity and relapse patterns following CAR-T cell therapy in EM B-ALL.

## Introduction

The introduction of targeted immunotherapy has expanded salvage options for patients with chemorefractory B-cell acute lymphoblastic leukemia (B-ALL) (1–5). With the US Food and Drug Administration approval of CD19-specific chimeric antigen receptor T cell (CAR-T) therapy, blinatumomab and inotuzumab ozogamicin, sequential targeted therapies are being delivered with increasing frequency to patients with B-ALL. Although CD19-CAR-T cells penetrate the blood brain barrier (6–10), antibody-based agents such as blinatumomab and inotuzumab do not (1, 11). The ability of these agents to circulate and access non-central nervous system (CNS) extramedullary disease (EM) sites is varied and remains incompletely described in children, adolescents and young adults (CAYA). There is evidence that CAR-T is effective at treating EM, with survival outcomes in CAYA with B-ALL EM comparable to patients with only medullary disease (6, 12). While EM had been associated with failure of blinatumomab in adults with relapsed ALL (13, 14), a Children’s Oncology Group (COG) study of standard chemotherapy versus standard plus blina for low risk relapsed ALL revealed superior outcomes with the addition of blina for patients with BM+/- EM disease but no benefit for patients with isolated EM (15). Adult studies have indicated that inotuzumab may aid with EM debulking in relapsed/refractory ALL (16, 17).

With increasing treatment options for relapsed/refractory leukemia patients, novel resistance patterns are becoming unmasked. A study of 180 relapsed refractory heavily pretreated patients referred for CAR-T identified ~21% of patients harbored non-CNS EMD (18). It remains unclear if distribution of disease or sites of relapse diverge following sequential immunotherapy, as compared to standard chemotherapy. Bone marrow aspiration and/or biopsy and lumbar puncture remain diagnostic standard of care for relapsed/refractory B-ALL, however do not adequately assess non-CNS EM. While there have been reports suggesting the utility of positron emission tomography (PET) imaging for patients with relapsed/refractory disease (19–24), this is not standard in the relapsed/refractory setting.

Here we describe 2 cases of patients with relapsed/refractory B-ALL and prior exposure to antigen-targeted immunotherapy who presented for CD19- or bi-specific CAR-T cells. On presentation, both patients had minimal disease burden measured by next-generation sequencing (NGS) minimal residual disease (MRD) on bone marrow without additional known sites of disease involvement. PET imaging was performed on both patients for distinct reasons prior to CAR-T infusion. In case 1, imaging was performed to rule out infection, while in case 2, imaging was performed in context of a broader work-up to evaluate new neurological symptoms thought to be related to chemotherapy-toxicity. Both patients showed evidence of non-CNS extramedullary disease on PET imaging that changed clinical management. These cases add to a developing body of literature that suggests a role for advanced imaging in EM assessment in relapsed/refractory B-ALL patients with prior exposure to antigen-targeted immunotherapy.

## Case one

Case one was an 8-year-old male with late relapsed B-ALL at 38 months from diagnosis who presented to our institution for relapse management. He received re-induction chemotherapy and bridging therapy with blinatumomab followed by tisagenlecleucel (CD19-specific CAR-T cells).

The patient was originally diagnosed with National Cancer Institute (NCI) standard risk CNS 1 pre-B cell ALL. FISH was notable for 11q23 (MLL) deletion and gain of 21q22 (RUNX1). He was treated as per COG AALL0932. He presented with relapsed B-ALL with 5% peripheral lymphoblasts <3 weeks after end of therapy. He was admitted and received chemotherapy re-induction with vincristine, doxorubicin, dexrazoxane, venetoclax, rylaze, and intrathecal methotrexate, hydrocortisone, and ara-c. He developed high fevers in context of neutropenia, causing disruption of chemotherapy. Extensive infectious workup demonstrated Rothia bacteremia, systemic herpes simplex virus (HSV), and disseminated candidal fungal infection (lungs, skin, CNS, eyes). He received prolonged treatment with broad-spectrum antibiotics, acyclovir, amphotericin, and voriconazole with resolution of his Rothia and HSV and improved control of his fungal infection. He was determined ineligible for allogeneic-hematopoietic stem cell transplantation (HSCT) at this time due to extensive infectious risk. Due to chemotherapy intolerance and infectious complications, blinatumomab was initiated as a bridge to CAR-T. He achieved a flow MRD negative response after 2 cycles of blinatumomab, underwent apheresis and tisagenlecleucel product was manufactured. While awaiting CAR-T cell treatment, he developed low level bone marrow involvement with 0.01% blasts in bone marrow by flow cytometry and 0.66% by deep sequencing NGS. The plan at that time remained to proceed to tisagenlecleucel treatment.

**Figure 1:**
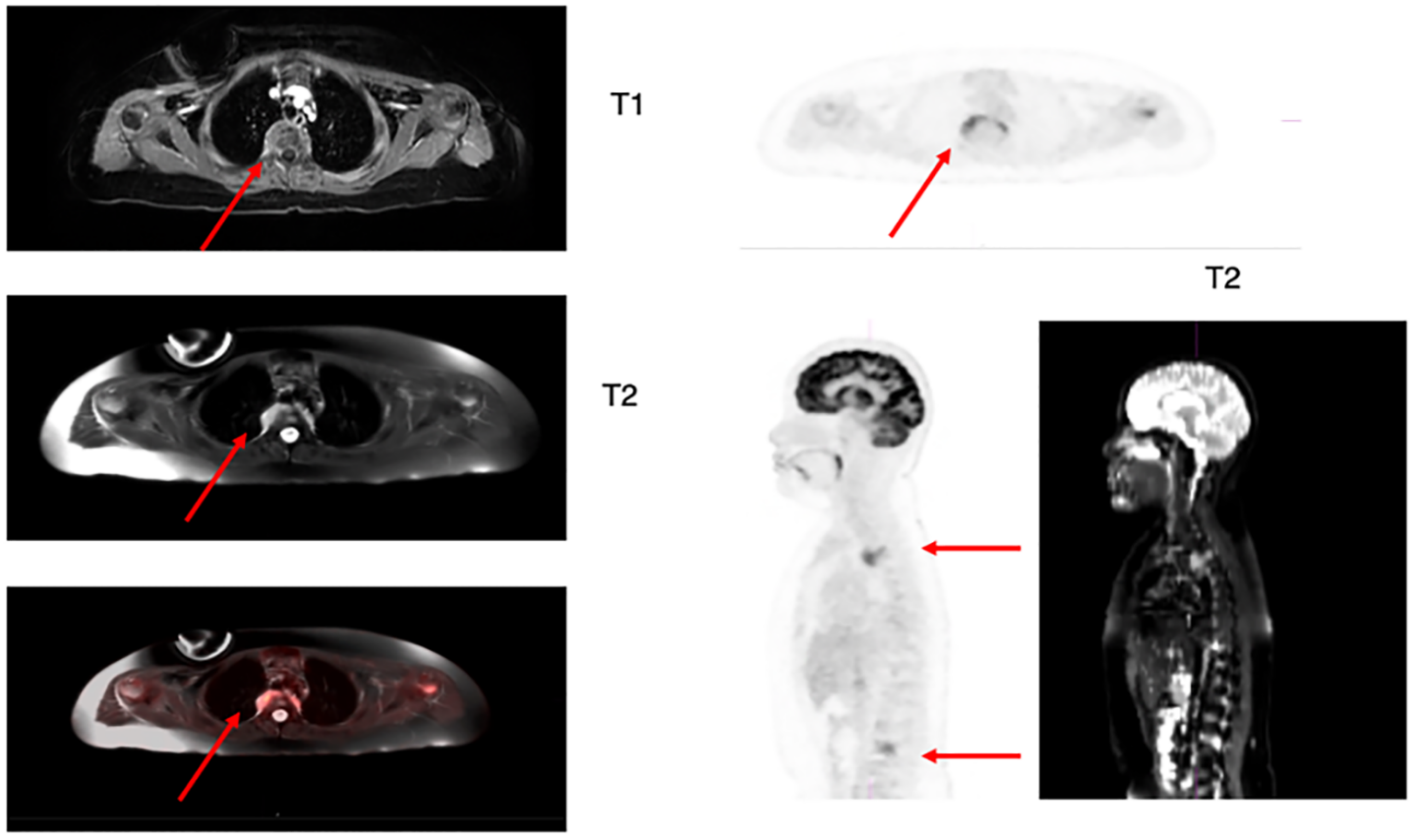
Axial and sagittal 18-FDG PET/MRI T1 and T2 weighted images showing hypermetabolic focal uptake in T2-T4 level and L3-L4 level. The MRI shows abnormal increased signal on axial view of the right posterior side of thoracic vertebral level with corresponding increased FDG activity on the PET scan images (arrow). The lumbar lesion is best identified on the sagittal views as increased metabolic activity in the lumbar spine on 18F-FDG PET and increased signal more focally in the spine in the areas of focal increased FDG activity (arrows).

Due to his profound infectious history, a work-up to exclude active infection was performed prior to initiating lymphodepletion (LD) and CAR-T therapy. Chest CT with contrast revealed previously unknown non-CNS EM with a thoracic spine lesion. Diagnostic full spine MRI with and without contrast showed multilevel leukemic involvement at C7, T3, T9, T10, L2 and L3, an enhancing soft tissue lesion T2-T4, involving right T3-T4 neuroforamen, and a right L3 lateral prevertebral soft tissue lesion involving right L3-L4 neural foramen (**Figure 1**). Due to concern for post-infusion CAR-mediated focal inflammation and associated risk for spinal cord compression, patient received radiation with 800cGy to the lumbar and thoracic spine prior to LD. He tolerated radiation and LD followed by tisagenlecleucel. He achieved medullary remission with undetectable disease by deep sequencing MRD. His spinal lesions showed initial pseudoprogression at 1month after CAR-T and then complete radiographic remission at 2 months. By this time, his fungal infection resolved and he was deemed a candidate for HSCT, which was pursued for consolidation of remission. The patient received an allogenic matched sibling donor transplant approximately 4 months after his tisagenlecleucel infusion. As of day 180 status post HSCT, his deep sequencing MRD was negative and donor chimerism was 98-100%. The presence of EM disease in this patient changed his treatment course, yet its detection was unmasked as an incidental finding during an infectious work-up, and not as an evaluation of EM disease burden.

## Case two

Case two was a 16-year-old female with multiply relapsed B-ALL and prior treatment with blinatumomab, inotuzumab, and allogeneic HSCT. Due to heavy pre-treatment, patient was referred to our institution for a Phase I clinical trial using bispecific CD19/CD22 CAR-T cells.

The patient was initially diagnosed with NCI high risk B-ALL with iAMP21 by FISH and positive KRAS p.A18D. She was enrolled on COG AALL1732. During maintenance cycle 6, the patient presented with an isolated bone marrow relapse in setting of poor adherence to oral chemotherapy (reflected in purine metabolites throughout maintenance). She received multiple lines of treatment, including blinatumomab, with progressive disease. She achieved a complete remission after inotuzumab with no disease detected on deep sequencing NGS MRD and eventually received a matched sibling donor HSCT with a conditioning regimen of Busulfan, Fludarabine, Clofarabine, rATG. Twenty-eight days after transplant, bone marrow assessment revealed positive deep sequencing MRD, and 2 weeks later was positive for disease by flow cytometry. For this, she required rapid withdrawal of immunosuppression with resultant gut GVHD treated with steroids. A few months later, the patient presented with a frank ocular relapse that necessitated treatment with steroids and localized radiotherapy. She subsequently received ruxolitinib for both GVHD and possible ALL treatment and achieved disease control with only low-level clones detected by NGS.

The patient was referred to our center for treatment with bispecific CD19/CD22 CAR-T treatment approximately 6 months after HSCT. Bone marrow NGS-MRD at that time increased from 1 to 100 clones per million cells (0.0001 to 0.01%) over 2 months, however she had no other established sites of B-ALL involvement. In the context of persistent low-level disease, the decision was made to pursue CAR-T therapy as a bridge to planned alpha beta depleted haploidentical HSCT. Due to prior exposure to both CD19 and CD22-monospecific agents, she was referred for bispecific targeting on clinical trial. The patient was enrolled on a single-institution bispecific CD19/CD22 CAR-T cell trial at dose level 3 and 10x10e6 CAR T cells/kg were manufactured for her.

**Figure 2:**
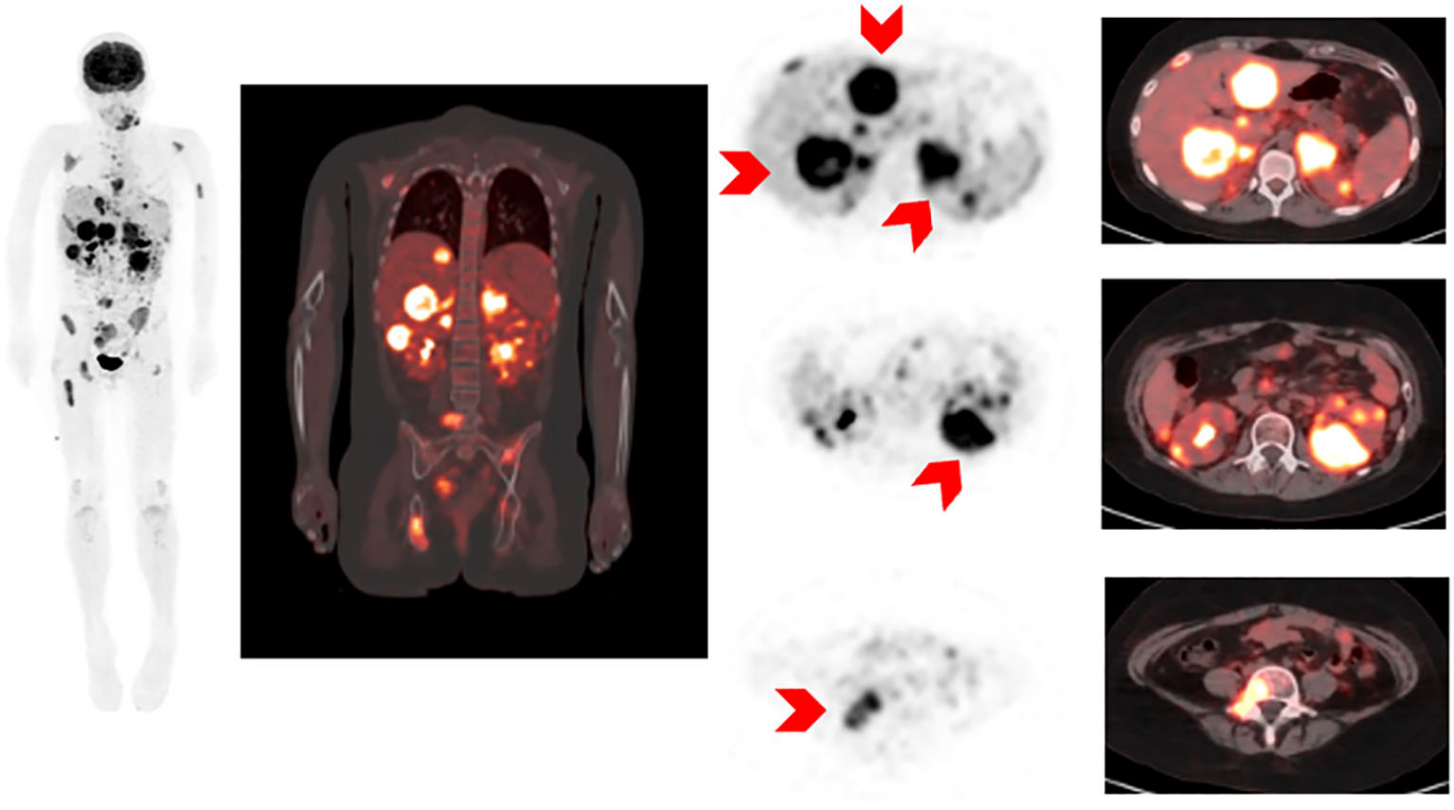
18F-FDG PET/MRI MIP (maximum intensity projection image) and selected axial images of 18-FDG PET and fusion PET images and MRI images showing diffuse abnormal 18F-FDG activity involving liver, kidneys, retroperitonal nodes, bowel and peritoneal implants, bony pelvis.

Prior to CAR-T infusion, the patient developed a right facial nerve palsy. An MRI Brain with and without contrast was overall unrevealing, although right greater than left auditory canal enhancement was noted and thought to be stable from prior. The patient was not known to have CNS disease involvement and cerebrospinal fluid was benign at this time without blasts (CNS1). Her facial nerve palsy was initially attributed to methotrexate-related encephalopathy. Because she had multiple relapses and new CNS symptoms, she underwent expanded disease work-up. PET-MRI demonstrated gross extramedullary disease with extensive involvement of the bones, lymph nodes, liver, adrenal glands, kidneys, GI tract, and peritoneal implants (**Figure 2**). These findings were consistent with extensive EM leukemic infiltrates and represented significantly higher disease burden than previously expected based on deep sequencing MRD in her marrow. Given newly-established higher disease burden, the patient was treated with low dose cytarabine as a bridge to the CAR-T infusion.

Despite bridging therapy, the patient demonstrated progressive disease in the bone marrow with 51.7% blasts by morphology on her pre-CAR-T infusion bone marrow. The patient initially enrolled on dose level 3 on trial. However, due to concerns for CAR-mediated toxicity in the setting of extensive leukemic burden and minimal dose level 3 toxicity data, an Expanded Access request was approved to instead give dose level 2 (3x10^6^ CAR T cells/kg) of CD19/CD22 CAR-T cells, a dose with prior established tolerability. The patient received a 3-day bridge with prednisone, followed by LD chemotherapy and received 3 million CAR-T cells/kg. Despite lower dosing, the patient experienced grade 1 cytokine release syndrome and immune effector-cell-associated hemophagocytic lymphohistiocytosis, complicated by grade 4 hepatic necrosis, respiratory insufficiency requiring positive pressure support and intra-abdominal hemorrhage requiring vascular embolization. Toxicities were managed as per American Society for Transplantation and Cellular Therapy and institutional standards with tocilizumab, anakinra and high-dose steroids. Day 28 bone marrow aspirate confirmed the presence of progressive disease with 72% lymphoblasts. She was ultimately admitted to hospice care and died on day 38 post-CD19/CD22 CAR of refractory B-ALL.

## Discussion

Antigen-targeted therapies have ushered in a new era for treating B-ALL. While treatment options for relapsed/refractory disease have expanded, the efficacy, distribution, and adverse effects of antigen-targeted therapies on non-CNS EM are being established. Further, there is no standard screening for non-CNS EM in relapsed/refractory patients who have had exposure to these agents. We describe two cases in which the findings of subclinical EM in relapsed refractory B-ALL patients significantly effected management prior to CAR-T infusion. In case one, findings were discovered during an infectious work-up. In case two, imaging was prompted by neurologic symptoms presumed to be chemotherapy-mediated, but revealed gross EM disease. In both cases, management was significantly altered due to concern for CAR-T-mediated toxicities with EM. In case one, radiation therapy was pursued prior to CAR-T. In case two, despite enrollment on dose level 3, CAR-T dosing was reduced to dose level 2, where tolerability was previously established. These cases contribute to a growing body of evidence raising awareness of subclinical EM in relapsed B-ALL in the era of targeted therapy and suggest a role for formal prospective imaging studies pre- and post-interventions to establish accurate disease staging and response assessments.

With increasing use of antigen-targeted therapies, it is crucial to establish relapse patterns post-targeted therapy and interrogate if distinct anatomic sites are inaccessible to these therapies, and thus serve as sanctuary sites and potential harbingers of relapse. Multiple retrospective studies provided evidence that while CD19, CD22, and CD19/22 combined CAR-T have activity against EM in relapsed B-ALL, these agents may be less effective in EM compared to medullary relapse (6, 12, 18, 25). Reports have similarly indicated that inotuzomab may have a role in debulking non-CNS EM or in combination with standard chemotherapy but is likely insufficient monotherapy for long term event free survival (16, 17). Studies of blinatumomab in patients with relapsed/refractory B-ALL have shown mixed findings regarding its utility in this setting. Aldoss et al. found that a history of non-CNS EM involvement predicts an inferior response to blinatumomab in a retrospective analysis of relapsed adult ALL patients (13, 14). Hogan et al. in their report on COG AALL1331 found that blinatumomab significantly improved survival in children and young adults with combined medullary and EM relapsed B-ALL, but gave no significant survival advantages for patients with isolated EM relapse treated with blinatumomab (15). Incorporating standard assessments and descriptions of EM trafficking and relapse patterns in studies with these agents will be fundamental in establishing the anatomic distribution and potential sanctuary sites from these agents.

With this, standardized assessment of EM in relapsed/refractory B-ALL may have an essential role in establishing accurate disease staging for these patients. Both cases reported here describe patients who were heavily pretreated and had exposure to targeted immunotherapies. The extent of their EM disease was not appreciated prior to PET-MRI and significantly altered their treatment courses. This adds to a growing number of case reports and retrospective studies in which PET imaging was essential in assessing leukemic EM (18, 20–25). We advocate that additional prospective studies should be undertaken to better describe the utility of PET imaging in patients with relapsed/refractory B-ALL and prior antigen-targeted therapies. Physicians should also consider incorporating imaging or functional imaging into the diagnostic work-up for complex multiply-relapsed patients as indicated in the setting of commercial targeted-therapy. Regarding the type of PET imaging, we see benefits to both PET-MRI and PET-CT. While PET-MRI is the standard at our institution and exposes patients to less radiation, PET-CT is more widely accessible.

Knowledge of EM can affect treatment and toxicity mitigation prior to CAR-T administration. It has been previously established that CAR-T directed against B-ALL has a unique set of toxicities in CNS disease (9, 10). There have been descriptions of inflammation and pain at the sites of EM disease in the kidneys, orbit, breast, and lymph nodes associated with CAR-T therapy as well as pleural effusions and new oxygen requirements associated with pleural EM (18, 26). A single case-report of CAR-mediated bilateral retinal detachment and vision-loss in a child with optic nerve and retinal leukemic infiltrates highlights the severity of possible functional damage in event of CAR-T cell-mediated inflammation local to sites of EM (27). Although these reports are limited in that they describe a small number of patients, were not conducted prospectively, and lack standard consensus management guidelines once EM is detected, each case provides valued insight on the side-effect profile of CAR-T in EM B-ALL. In case one, we describe a patient who had multifocal bony and extramedullary B-ALL entering his spinal canal and abutting his dura, and another with evidence of multi-organ disease involvement on imaging. Toxicities of CAR-mediated focal inflammation when B-ALL EM disease is present within the spinal canal have yet to be described. Due to concern for CAR-mediated inflammation and para-spinal edema and lack of data, this patient received pre-CAR radiation in an effort to mitigate toxicity. The patient in case two received a lower dose of CAR-T cells than originally planned due to extensive EM and bone marrow involvement and safety concerns, due to prior literature supporting increased toxicities with increased disease burden (28). It remains unknown how the patients in this series would have responded without modification in their treatment plans. These cases underscore the need to comprehensively establish baseline sites of B-ALL involvement prior to CAR-T cell therapy to facilitate toxicity preparedness and mitigation.

It is likely that a subset of patients with multiply relapsed B-ALL have sub-clinical sites of EM disease that are underrecognized. Although CAR-T can serve as a valued effective salvage option for chemotherapy-refractory B-ALL, the clinical impact of subclinical EM disease in the pre-CAR setting is not yet clear. We therefore highlight the importance of imaging studies for systematic EM disease evaluation at baseline and in the context of relapse post-immune-targeted therapy. We anticipate that formal study of distributive patterns at time of relapse in the setting of targeted immunotherapy will help to establish updated diagnostic and management standards.

## Data Availability

The original contributions presented in the study are included in the article/supplementary material. Further inquiries can be directed to the corresponding author.
